# Inertial measurement data from loose clothing worn on the lower body during everyday activities

**DOI:** 10.1038/s41597-023-02567-4

**Published:** 2023-10-17

**Authors:** Udeni Jayasinghe, Faustina Hwang, William S. Harwin

**Affiliations:** Biomedical Engineering Section, University of Reading, RG6 6DH Reading, UK

**Keywords:** Biomedical engineering, Quality of life

## Abstract

Embedding sensors into clothing is promising as a way for people to wear multiple sensors easily, for applications such as long-term activity monitoring. To our knowledge, this is the first published dataset collected from sensors in loose clothing. 6 Inertial Measurement Units (IMUs) were configured as a ‘sensor string’ and attached to casual trousers such that there were three sensors on each leg near the waist, thigh, and ankle/lower-shank. Participants also wore an Actigraph accelerometer on their dominant wrist. The dataset consists of 15 participant-days worth of data collected from 5 healthy adults (age range: 28–48 years, 3 males and 2 females). Each participant wore the clothes with sensors for between 1 and 4 days for 5–8 hours per day. Each day, data were collected while participants completed a fixed circuit of activities (with a video ground truth) as well as during free day-to-day activities (with a diary). This dataset can be used to analyse human movements, transitional movements, and postural changes based on a range of features.

## Background & Summary

Inertial measurement units (IMUs) are increasingly popular as wearable sensors in the healthcare^1–3^ and sports sectors^4–6^. In healthcare, wearable sensors offer a way to capture data about people’s everyday activities easily and in an economical way, both within and outside clinical environments^7^. Mosenia *et al*.^8^ noted that wearable sensors in health monitoring can reduce the costs of long-term care in hospitals. These sensors can be used in different types of movement analyses such as human posture classification^9,10^, activity classification^11,12^, gait analysis^13,14^, transitional movement analysis^15,16^, sleep monitoring^17,18^ and falls detection^9,19^.

While a number of studies investigate the use of a single wearable sensor (e.g. on the wrist or on the lower back), increasing the number of sensors can help with improving the accuracy of monitoring systems and capture a more complete view of the body’s movements. Though multiple sensors increase the accuracy of human activity recognition^20–24^ (HAR), putting on and wearing multiple sensors can be a tedious or laborious task for the wearer. There are also potential challenges with ensuring the sensors are placed in appropriate locations and orientations. One approach to improving the process of wearing multiple sensors is to embed these sensors into clothing^25–28^. Most previous studies have experimented with tight-fitting clothes^29–32^ to help ensure the sensors stay close to the limbs without moving during data collection. In a healthcare context where tight-fitting clothes may not be appropriate nor desirable, attaching multiple sensors to loose-fitting, everyday clothing offers comfort and convenience^28,33,34^, without the burden of needing to strap on sensors one-by-one and adjusting them. This research investigates sensors in loose-fitting, everyday clothing so the wearer can have them on for longer periods in a comfortable way. This approach reduces the time that it takes to put on multiple sensors, as the sensors are embedded in the clothing and putting on multiple sensors becomes a matter of getting dressed. Further, by embedding the sensors in the clothing, the burden of ensuring the correct positioning and orientation of the sensors is reduced or even eliminated for the wearer. This opens up opportunities to make these measurements in previously hard to reach populations and environments e.g. long-term healthcare monitoring.

There are already publicly-available databases of Human Activity Recognition (HAR)-related wearable sensor data. These include data collected from a waist-mounted smartphone with accelerometer and gyroscope sensors^35–38^, a waist-mounted IMU^39^, an ankle-mounted IMU with a stretch sensor^40^ and 17 Magnetic, Angular Rate, and Gravity (MARG) sensors mounted on the head, shoulders, chest, arms, forearms, wrist, waist, thighs, shanks, and feet^41^. Further, databases are available for gait analyses such as Luo *et al*.’s^42^ study with 6 body-worn IMUs, Lencioni *et al*.’s^43^ study using camera motion, force plates and electromyography (EMG) and Loose *et al*.’s^44^ study using Xsens sensors on both feet, shanks, thighs and pelvis.

The present database has loose clothing-embedded IMU data from the lower body, alongside video recordings and diaries as ground truth data. The data were recorded from semi-natural activities i.e. a video-recorded pre-defined set of activities (standing, sitting, lying down, sitting with legs outstretched, walking, climbing up and down stairs - approximately 20 minutes in total) and participants’ usual day-to day activities during the rest of the day along with diary data for 5 to 8 hours. Data were collected from five healthy participants for between 1–4 days per person, for a total of 15 participant-days’ of data. To our knowledge, this is the first published database consisting of data collected from loose clothing-embedded IMUs. This dataset is likely to be of interest to researchers studying human postures and movements in natural settings, particularly that the sensors are worn unobtrusively in loose-clothing rather than on the body and also that the data includes measurements of the waist, thigh and ankle on both the left and right sides.

We have previously published a paper^45^ based on this dataset^46^ where a posture classifier was implemented using a single feature (the inclination angle estimated from the accelerometer data) from three sensors (waist, thigh and ankle). Four postures (standing, sitting, lying down and sitting on the floor with legs outstretched) were classified with a high level of accuracy, demonstrating that the data from the sensors embedded in clothing can be used productively in posture classification. With this earlier paper, we published some of the processed data, specifically the inclination angles from a subset of the sensors. The aim of the present paper is to make available a more detailed dataset from the clothing on the lower-body, which includes data from six IMUs (accelerometers, gyroscopes, and magnetometers) and from a wrist-worn sensor, along with videos, diaries, and annotations of the activities, which we anticipate will enable further research and analysis.

## Methods

### Materials

The data^46^ presented in this paper were collected as part of a larger dataset from sensors in the clothing on both the upper and lower-body, as well as a wrist-worn sensor (not attached to clothing). Here, we present the data from the lower body only; We are planning to publish the upper-body data after they have undergone further cleaning and analysis. Once available, they can be combined with the lower-body data from the present publication for a more comprehensive analysis.

**Fig. 1:**
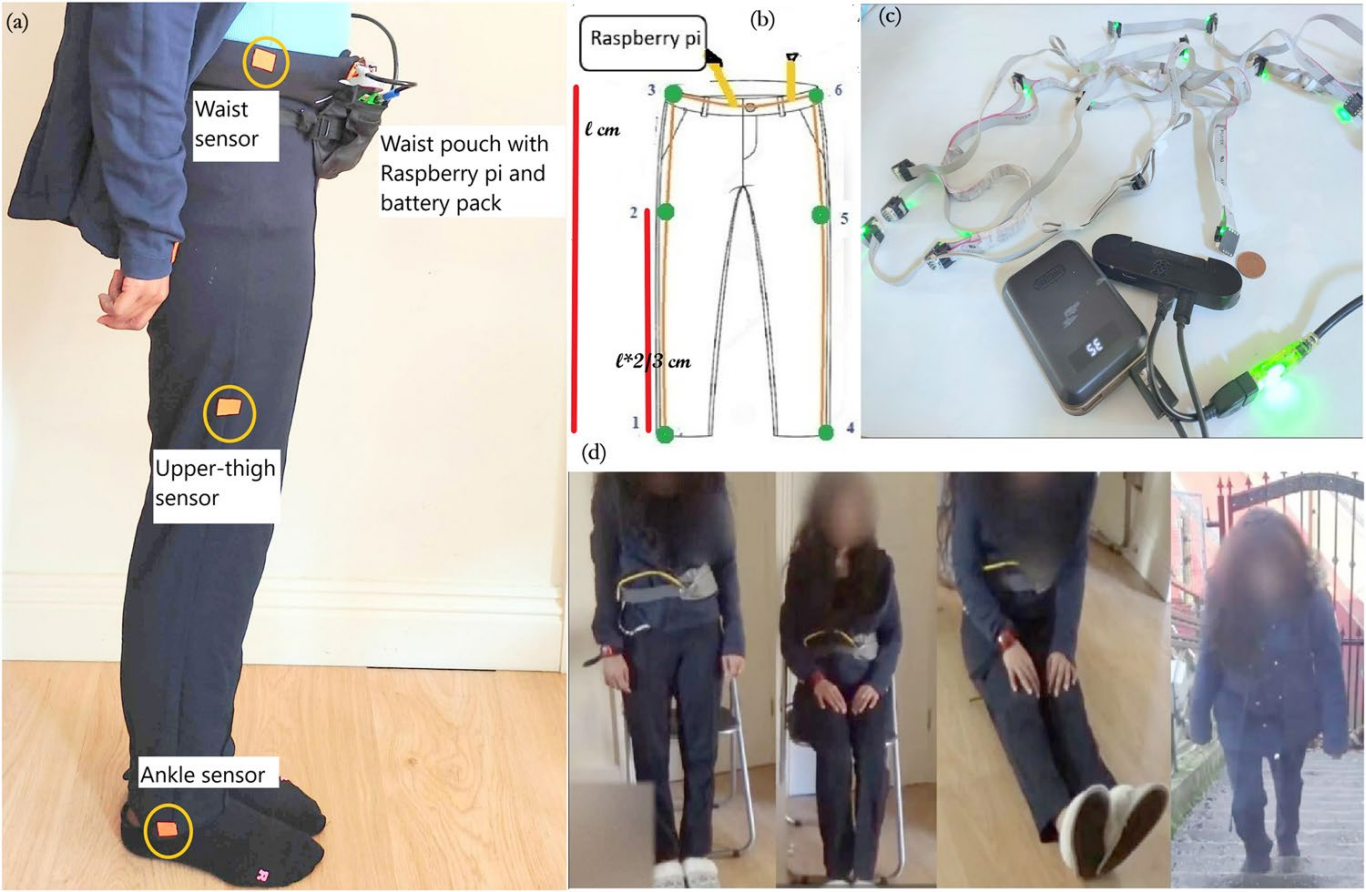
Sensor placement on the clothing (**a**) and set up of the sensor strings (**b**). The sensor placements are indicated with stickers in (**a**). The measurements used to position the sensors are shown in (**b**), where *l* is the leg length. Ankle sensors were placed near the hem of the trousers (sensors 1 and 4 as marked in (**b**)). The thigh sensors were placed at *l* ×2/3 (two-thirds of *l*) above the ankle sensor (sensors 2 and 5 as marked in (**b**)). The IMUs connected to the battery-powered Raspberry Pi are shown in (**c**), where a one-penny coin is included for scale. The present paper focuses on only the data from the 6 sensors on the lower-body. Screenshots from video recordings for some of the actions are given in (**d**).

The sensing system in the clothing consisted of 12 IMUs (based around the Bosch Sensortec BMI160 smart IMU), all using a differential serial bus, connected via flat ribbon cable forming a “sensor string”. The 12 bespoke sensors were approximately 15 × 12 × 7 mm each (see Fig. 1b) and weighed 18 g in total while the inter-connecting cables weighed 146 g. The string was connected to a Raspberry Pi where the data were stored. The battery pack enabled continuous mobile data collection for more than 12 hours (10000 mAh output: 5 V, 2.1 A). Data were sampled at 50 Hz. The range of the accelerometers was + /− 16 g with 12-bit resolution. The BMI160 IMU includes a gyroscope with a range of 1000 degrees per second and magnetometer, which were also recorded along with accelerometer readings. Since accelerometer, magnetometer, and gyroscope data were all recorded from each sensor, a time division multiplexing bus protocol running at 500 K baud was used.

The 12 IMUs were positioned in the clothing so that there were three sensors along the lateral side of the upper limbs (wrist, upper arm, and shoulder/neck) and lower body (ankle, thigh, waist), on both the left and right sides (Fig. 1).

The sensor placement was informed by recent analyses of sensor placements^9,11,13,47–50^. Prior work has suggested placing a sensor on the ‘thigh’ for classifying postures and some physical activities^11^. As the present data collection included cyclic movements such as walking and climbing up and down stairs, sensors were also placed on both ankles/ lower-shanks^13^. As most of the published posture classifiers were based on waist/ chest data, two sensors were placed on the waist^9,47,48^. According to the sensor placements suggested by Gemperle *et al*.^49^, two sensors were positioned near the rear collar area and another two on the upper arms. Finally, as the wrist is the most common place to mount a wearable device^50^, two sensors were also positioned on the wrists.

To attach the sensors to the clothes, the sensors were taped securely along the seams of the clothes in the chosen positions as shown in Fig. 1b and cotton bias binding was taped on top of the sensor string using double-sided tape for fabric. In this way, the sensors were not outwardly visible and also not in contact with the skin. That helped to make the outfit with sensors more comfortable for the wearer. Participants were asked to rate the physical comfort of the clothes on a 7-point scale from very uncomfortable (1) to very comfortable (7). Their responses ranged from 4 to 6.

In addition to the clothing-worn sensors, an Actigraph, device was strapped onto the wrist of the dominant hand of the participant as a reference, body-worn sensor. The Actigraph sampling rate was also set to 50 Hz.

### Data collection procedure

Five healthy participants (age range: 28–48 years old; 3 males and 2 females) took part in this study. Each person selected a pair of trousers and a hoodie jacket in their usual size, and the researcher attached the sensors to the clothes. Four participants wore cotton-blend fleece jogging trousers, and one wore loose cotton slacks. (One of the male participant’s trousers were baggy at the thigh, compared to the other participants’ trousers.) The sensor readings can be affected depending on the looseness of the clothing, as discussed in another study^51^. Participants were asked to wear the clothes over multiple days for 5–8 hours per day of data collection. Participants gave written informed consent for these data to be made publicly available for use by others, and this was approved by the ethics committee of the School of Biological Sciences, University of Reading, UK (SBS 19- 20 31 and SBS 21- 22 18). The study was conducted in accordance with this approved protocol and the relevant guidelines and regulations.

The Raspberry Pi and the battery pack were kept in a pouch on the waist of the participant. Once the Raspberry Pi was powered on, it started recording data. Further, to check that the data were being recorded, the Raspberry Pi could be accessed with a mobile phone via SSH (secure shell). Figure 1a shows a participant with the clothing-embedded sensors with the Raspberry Pi on the waist. The sensors were not visible from the outside of the clothing, other than the waist bag with the Raspberry Pi.

On each day of data collection, participants were asked to perform a set of predefined activities, and these activities were video-recorded using a camcorder by a third person as shown in Fig. 1d to provide a ground truth. Ground truthed data were recorded for the following set of activities (in order):

1. Standing still for 2 minutes
2. Sitting (on a chair) for 2 minutes
3. 5 cycles of raising the legs while sitting down
4. 5 Sitting-to-standing cycles
5. Walking back and forth for 2 minutes
6. Climbing up and down stairs for 2 minutes
7. Lying down for 1–2 minutes
8. Sitting on the floor with legs outstretched for 1–2 minutes

After the predefined activities, the participants were asked to continue with their usual activities for the rest of the day (5 to 8 hours). During that time, the participants were requested to keep a diary of their activities and the times of those activities. Participants were given an electronic diary template to keep track of the start time, end time and the description of the activity with a sample activity list (standing, walking, sitting, going up and down stairs, running) plus space to add activities that were not on the sample list. Some participants used the electronic template whereas others elected to record their activities on paper or in text files, mainly noting the start time for each activity (the end time was then taken to be the start time of the next activity).

To create the diary files included with the dataset, participants’ notes were transcribed so that all the files were in a similar format, and additional information was added, i.e. the number of videos, number of missing data segments, and the start and end points for the activities that were video recorded.

This data repository consists of data from 15 days across five participants (see Table 1), with each participant contributing between 1 and 4 days’ of data.

**Table 1:** Data catalogue.

	Day (name)	Activities in ground truth video									Start time	End time	Duration	Notes
		Standing	Sitting	Walking	Climbing up stairs	Climbing down stairs	5 leg raises	5 sit-to stands	Lying down	Sitting on the floor				
P1	Day 1 (P1D1)	✓	✓	✓	✓	✓	✓	✓	✓	✓	09:40	18:20	8 h 40 m	*Jogging trousers (baggy)*
	Day 2 (P1D2)	✓	✓	✓	✓	✓	✓	✓	✓	✓	11:15	19:20	8 h 05 m	
	Day 3 (P1D3)	✓	✓	✓	✓	✓	✓	✓	✓	✓	11:30	20:00	8 h 30 m	
P2	Day 1 (P2D1)	✓	✓	✓	✓	✓	✓	✓	✓	✓	10:50	17:10	6 h	*Loose cotton slacks* Weekend
	Day 2 (P2D2)	✓	✓	✓	✓	✓	✓	✓	✓	✓	12:15	18:45	6 h 30 m	Weekend 30 mins data missing
	Day 3 (P2D3)	✓	✓	✓	✓	✓	✓	✓	✓	✓	12:15	17:20	5 h	10 star jumps, 3 burpees 20 mins data missing
	Day 4 (P2D4)	✓	✓	✓	—	—	✓	✓	✓	✓	10:10	15:50	~6 h	one hour data missing
P3	Day 1 (P3D1)	✓	✓	✓	✓	✓	✓	✓	✓(1 min)	✓(1 min)	07:50	12:30	~4 h	*Jogging trousers* 1 hour data missing
P4	Day 1 (P4D1)	✓	✓	✓	✓	✓	✓	✓	✓(1 min)	✓(1 min)	09:10	14:55	~6 h	*Jogging trousers*
	Day 2 (P4D2)	✓	✓	✓	✓	✓	✓	✓	✓(1 min)	✓(1 min)	09:40	16:56	6 h 30 m	
	Day 3 (P4D3)	✓	✓	✓	✓	✓	✓	✓	✓(1 min)	✓(1 min)	08:40	15:51	7 h	
P5	Day 1 (P5D1)	✓	✓	✓	✓	✓	✓	✓	✓	✓	14:00	18:40	~5 h	*Jogging trousers*
	Day 2 (P5D2)	✓	✓	✓	✓	✓	✓	✓	✓	✓	14:00	19:10	5 h	
	Day 3 (P5D3)	✓	✓	✓	✓	✓	✓	✓	✓	✓	10:20	16:10	6 h	
	Day 4 (P5D4)	✓	✓	✓	✓	✓	✓	✓	✓	✓	10:22	16:10	~6 h	

There are two minutes of data for standing, sitting, walking, climbing up/ down stairs, lying down and sitting on the floor (marked with a ‘✓’) unless otherwise indicated (‘−’ indicates missing data). The start time and the end time of the data collection at the end of each day are given in the table, along with special notes such as whether the data were collected on a weekend, if special activities were performed, and which type of trousers they were wearing and about the missing data.

### Data workflow

#### Data storing and decoding

Data from the IMUs were serialised onto a twisted pair RS485 bus using ‘base64’ and saved on the Raspberry Pi through the serial port. Once a participant had completed their part in the study, these files were transferred to a PC, decompressed and analysed in MATLAB. Following a data cleaning and alignment process the data were saved as MATLAB ‘MAT’ files.

#### Data cleaning

There were some signal losses owing to power supply issues during the data collection. Those points were identified by synchronising the dominant hand’s ‘wrist’ clothing-sensor data with the Actigraph data and replacing missing segments with zeros. Altogether 3% of data is missing from these ~102 hours of data. The primary reason for the missing data was cables becoming disconnected due to movements.

#### Pre-processing

All sensors used to collect data were individually calibrated against the magnitude and direction of the gravity vector so that a homogeneous transform matrix for each sensor could be calculated. This matrix then allowed corrections for scaling and axis orthogonality errors for each sensor.

Data were then processed to align the sensors to each limb as the orientation of the sensors inside the clothing was uncertain. Two rotation transforms were calculated to orient the data from each sensor relative to the presumed axis of the limb and then to the principal plane of movement of that limb. Thus the first rotation changes the data from the sensor frame {*S*} to an intermediate frame {*I*} and the second rotation changes the data from the intermediate frame to the final frame {*F*}.

The first rotation was applied to align the z-axis of the sensor to the direction of gravity (superior-inferior). Following the application of this rotation to the data, the z-axis of the intermediate frame {*I*} was closely aligned with the gravity vector $\underline{{\bf{g}}}$. A period when the participant was standing still and the limb could be assumed to be vertical was chosen from the data and *m* points were sampled. The rotation matrix ${}_{S}^{I}R$ was calculated by determining an angle and axis for the rotation. (Note the notation here indicates that vectors in the {*S*} frame were, after multiplication by ${}_{S}^{I}R$, the same vectors but now expressed in the intermediate {*I*} frame).

Since there is no movement during this ‘standing still’ period, the sensors collected *m* data vectors that represent ${}^{S}\underline{{\bf{g}}}{\simeq }^{S}{\underline{{\bf{a}}}}_{k}$ where 1 ≤ *k* ≤ *m*, i.e. the coordinates of the gravity vector in the sensor frame {*S*}. The magnitude of this vector should be approximately 9.81 *ms*^−2^ if the sensors are calibrated in metric units or 1 if calibrated in gravitational units. For convenience gravitational units are assumed for this section. Equation 1 calculates the average value of acceleration during period *m* from the individual measurements *a*_*j, k*_

1 $${}^{S}\underline{{\bf{a}}}={\left[\begin{array}{ccc}{}^{S}{a}_{1} & {}^{S}{a}_{2} & {}^{S}{a}_{3}\end{array}\right]}^{T}\quad {{\rm{where}}}^{S}{a}_{j}=\frac{1}{m}\mathop{\sum }\limits_{k=1}^{m}{a}_{j,k}\quad {\rm{for}}\quad j=1,2,3\quad {\rm{referring}}\;{\rm{to}}\;{\rm{the}}\;{\rm{x,}}\;{\rm{y}}\;{\rm{and}}\;{\rm{z}}\;{\rm{axes}}$$

This estimate can be readily converted to a unit vector that approximates ${}^{S}\underline{{\bf{g}}}$ in gravitational units using the ‘hat’ notation in Eq. 2.

2 $${}^{S}\underline{{\bf{g}}}\simeq {}^{S}\underline{\widehat{{\bf{a}}}}={}^{S}\underline{{\bf{a}}}/| \underline{{\bf{a}}}| ,\quad {\rm{where}}\quad | \underline{{\bf{a}}}| =\sqrt{{a}_{1}^{2}+{a}_{2}^{2}+{a}_{3}^{2}}$$

The first rotation converted ${}^{S}\underline{{\bf{g}}}$ to ${}^{I}\underline{{\bf{g}}}$ where it was assumed that ${}^{S}\underline{{\bf{g}}}\simeq {}^{S}\underline{{\bf{a}}}$. This was achieved by using the basis vector for the sensor z-axis $${}^{S}\underline{\widehat{{\bf{z}}}}={\left[\begin{array}{ccc}0 & 0 & 1\end{array}\right]}^{T}$$.

The axis of rotation $\underline{{{\bf{r}}}_{{\bf{1}}}}$ was chosen to be perpendicular to both ${}^{S}\underline{{\bf{a}}}$ and ${}^{I}\underline{\widehat{{\bf{z}}}}$ so could be estimated as

$$\underline{{{\bf{r}}}_{{\bf{1}}}}={}^{S}\underline{{\bf{a}}}\times {}^{S}\underline{\widehat{{\bf{z}}}}$$

($\underline{{{\bf{r}}}_{{\bf{1}}}}$ has the same elements in both the {*S*} and the {*I*} coordinate frames)

To work correctly as an angle axis representation $\underline{{{\bf{r}}}_{{\bf{1}}}}$ should be redefined to be a unit vector and this was done using Eq. 2.

The angle of the rotation was estimated from the dot product between ${}^{S}\underline{{\bf{a}}}$ and ${}^{{}^{S}}\underline{\widehat{{\bf{z}}}}$ since the definition of the dot product is

$${}^{S}\underline{{\bf{a}}}\cdot {}^{S}\underline{{\bf{z}}}=| {}^{S}\underline{{\bf{a}}}| \cos ({\theta }_{1})$$

If ${}^{S}\underline{{\bf{a}}}$ is the unit vector aligned with ${}^{S}\underline{{\bf{a}}}$, then *θ*_1_ could be computed simply as

$${\theta }_{1}=\mathrm{acos}({}^{S}\mathop{\hat{{\rm{a}}}}\limits_{\_}\cdot {}^{S}\mathop{\hat{{\bf{z}}}}\limits_{\_})$$

Both the angle and the axis were then available to compute the rotational transform using Rodrigues’ formula.One form of Rodrigues’ equation is shown in Eq. 3 where K is a skew symmetric matrix derived from ${\underline{{\bf{r}}}}_{1}$. This ‘K’ (Eq. 3) can be expressed with the elements of the ${\underline{{\bf{r}}}}_{1}$.

3 $${}_{S}^{I}R=I+\sin {\theta }_{1}K+(1-\cos {\theta }_{1}){K}^{2}$$

where $$K=\left[\begin{array}{ccc}0 & -{r}_{1}(3) & {r}_{1}(2)\\ {r}_{1}(3) & 0 & -{r}_{1}(1)\\ -{r}_{1}(2) & {r}_{1}(1) & 0\end{array}\right]$$ and *I* is the 3 × 3 identity matrix.

The first rotation matrix was thus calculated from Eq. 3 using the data from the individual sensor accelerometers. Thereafter the same rotational matrix was then applied to the gyroscope and magnetometer data and the data from the sensors converted to this intermediate frame.

After applying the first rotation, any movements of the limb in the sagittal plane can be used to reorientate the x and y axes to the final coordinate frame {*F*}. The z-axis remains the same for both the intermediate {*I*} and the final {*F*} coordinate frames. The concept was to choose the direction of the lowest principal component of acceleration as the direction for the final x-axis.

For this paper ‘sitting to stand’, ‘walking’ and ‘leg raising while seated’ were selected as movements that happen in the sagittal plane from the perspectives of the waist, thigh and ankle respectively. Data for each of these segments, once converted to the intermediate frame, was selected to define the second rotation from the intermediate to the final coordinate frame.

The second rotation was computed and applied to make sure that the sagittal plane motions (i.e. sitting to stand, walking and leg raising while seated) would be in the y-z plane of the final coordinate frame such that the y-axis aligns with the anterior-posterior direction in the sagittal plane and the x-axis with the medial-lateral direction perpendicular to the sagittal plane.

Multiple methods to identify the plane of principal movement are possible, for example, defining the plane-of-motion to be a plane perpendicular to the direction of minimum acceleration, or identifying a unit vector that aligns with any reasonably large angular velocity. However, the preference, in this case, was to use the same IMU sensor, the accelerometer, to estimate both rotational transforms. Suitable data segments with movements in the sagittal plane were selected from the accelerometer for each IMU sensor. The eigenvectors of the covariance matrix of the centred data segment give directions of maximum and minimum accelerations that align with the x and y-axis of the final frame. These Eigenvectors are known to be orthogonal and can be readily computed either directly as Eigenvectors or from the singular value decomposition of the segmented data.

The second rotation occurs around the z-axis of the intermediate frame, which will also become the z-axis of the final frame. The direction of the vector ${}^{I}\underline{\widehat{{\bf{m}}}}$ corresponding to the smallest singular value or smallest Eigenvalue was used to identify the axis orthogonal to the z-axis of the intermediate frame. This vector was assumed to be orthogonal to most movements in the sagittal plane.

After finding the axis of the lowest principal component in the intermediate frame (${}^{I}\underline{\widehat{{\bf{m}}}}$), the second axis (${}^{I}\underline{\widehat{{\bf{f}}}}$) (forward-backward acceleration) was confirmed by using vector cross product in Eq. 4 so that it was perpendicular to the axis of minimum acceleration.

4 $${}^{I}\underline{\hat{{\bf{f}}}}={}^{I}\underline{\hat{{\bf{z}}}}\times {}^{I}\underline{\hat{{\bf{m}}}}\quad {\rm{where}}{}^{I}\underline{\hat{{\bf{m}}}}={}^{I}\underline{{\bf{m}}}/| \underline{{\bf{m}}}| $$

Vectors $\underline{{\bf{m}}},\underline{{\bf{f}}}$, and ${}^{I}\underline{{\bf{z}}}$ were then associated with the directions of the x y and z axis of the final frame respectively and used to calculate the final rotation matrix ${}_{I}^{F}R$.

By using Rodrigues’ rotation formula again (as described in Eq. 3), a second rotation was applied (using Eq. 4 and Eq. 5) so that the transformed y-axis is aligned with the anterior-posterior direction and the transformed x-axis is aligned with the medial-lateral direction perpendicular to the sagittal plane.

5 $$\mathop{{{\bf{r}}}_{{\bf{2}}}}\limits_{\_}={}^{I}\mathop{\hat{{\bf{f}}}}\limits_{\_}\times {}^{I}\mathop{\hat{{\bf{y}}}}\limits_{\_}\,\,{\rm{where}}\,\,{}^{I}\mathop{\hat{{\bf{y}}}}\limits_{\_}=[0\,\,1\,\,0{]}^{T}\,\,{\rm{and}}\,\,{\theta }_{2}={\rm{acos}}({}^{I}\mathop{\hat{{\bf{f}}}}\limits_{\_}\cdot {}^{I}\mathop{\hat{{\bf{y}}}}\limits_{\_})$$

#### Annotation

To annotate the data, the videos were synchronised with the sensor data using ELAN software^52^. The start and end points for each different posture and activity were manually identified by the first author, and those segments were annotated and saved in a file.

## Data Records

The final labelled dataset is located at figshare^46^ and comprises 15 participant-days of data across the 5 participants, with 6 video-ground truthed activities per participant per day. The data are organised in folders with a naming convention of ‘PXDY’ where X is the participant ID and Y indicates the day of the data collection (e.g. P1D1- Participant 1 Day 1). All faces in the videos (both participant and bystander) were blurred using a combination of automated and manual methods. First, the videos were passed through a software tool that does face-blurring automatically. The videos were then manually checked, and if any visible faces remained, they were blurred manually. After face-blurring, the videos were sent to the participants again to confirm their comfort with having them published.

**Fig. 2:**
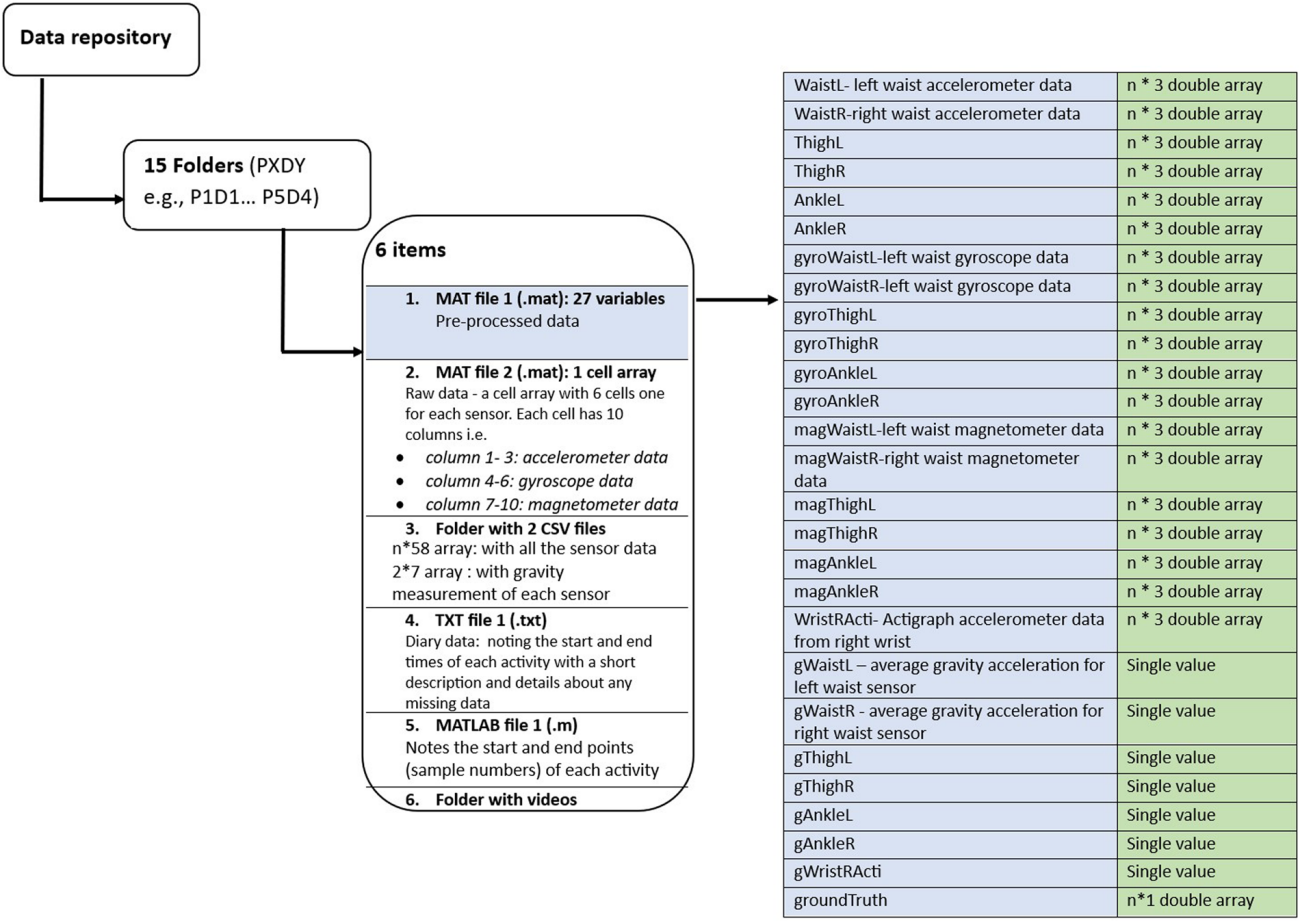
Data structure of the data repository. The repository contains 15 folders. Each folder contains 2 MAT files, a folder with two CSV files, 1 text file (diary data), 1 MATLAB file (video annotation file) and a folder with video files. The naming convention is ‘PXDY’ where X is the participant ID and Y is the day of the data collection (e.g. P1D1- Participant 1 Day 1).

Each folder contains 6 items i.e. Data structure of the data repository. The repository contains 15 folders. Each folder contains 2 MAT files, a folder with two CSV files, 1 text file (diary data), 1 MATLAB file (video annotation file) and a folder with video files.The detailed version of the folder structure is given in Fig. 2.

The file “PXDY.MAT” loads all the pre-processed data (orientation corrected) from each position/sensor along with the annotations (groundTruth). All the variable names are given in Table 2.

**Table 2:** Variable names for a full-day dataset, including all the data from the pre-defined activities as well as the “rest of the day activities” of a participant.

Sensor	Side	Variable name			
		Accelerometer data	Gyroscope data	Magnetometer data	Average gravity measured by the sensor
Waist	Left	WaistL	gyroWaistL	magWaistL	gWaistL
	Right	WaistR	gyroWaistR	magWaistR	gWaistR
Thigh	Left	ThighL	gyroThightL	magThighL	gThighL
	Right	ThighR	gyroThightR	magThighR	gThighR
Ankle	Left	AnkleL	gyroAnkleL	magAnkleL	gAnkleL
	Right	AnkleR	gyroAnkleR	magAnkleR	gAnkleR
Actigraph Wrist-worn	Dominant hand	WristRActi	—	—	gWristRActi
—	—	groundTruth: 1- standing, 2- sitting, 3- lying down, 4- sitting on the floor, 5- walking, 6- climbing up stairs, 7- climbing down stairs, 8- sit-to-stands, 99- not defined			

The Actigraph sensor has only accelerometer data (it does not have gyroscope and magnetometer data, as indicated by a ‘—’.)

The file “PXDYDiary.txt” has approximate start and end times for activities and a brief description of the activities. In addition to the diary entries, the file contains a description with details of the date, start and end times of the data collection and whether or not there are missing data (i.e. if there was a power failure).

From the diary data, the most common daytime activities of the participants were working at a computer while sitting at a desk or sitting on a sofa and walking indoors/outdoors. Occasionally, there were activities such as star jumps, driving, shopping, house chores (loading and unloading the washing machine, doing dishes, cooking), floss dance and burpees.

## Technical Validation

To validate the sensor information against “true” values requires a cumbersome measurement system such as a 3D optical motion capture system. This is not feasible when studying people’s everyday activities in natural environments. Instead, we conducted a visual inspection of the videos to assess whether there was a reasonable association between the sensor data (e.g. angles) and what was observed in the video. In this section, we present plots of the sensor data across a range of activities and discuss how they relate to the activities that were being performed.

**Fig. 3:**
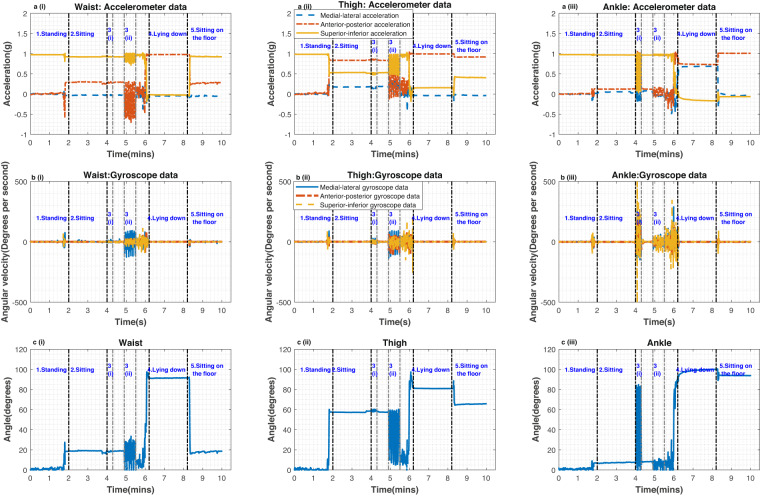
Sensor data and sensor to vertical angles for (1) standing, (2) sitting, (3i) - leg raises while sitting and (3ii)- sit-to stands, (4) lying down and (5) sitting on the floor with legs outstretched. The top (a(i)-a(iii)) and middle ((b(i)-b(iii)) plots show the accelerometer and gyroscope data respectively, for the waist, thigh, and ankle sensors. The bottom plots c (i) - c (iii) show the angles of each sensor with respect to vertical. These data were from the right side from the Participant 2 Day 2 dataset.

Figure 3a(i–iii),b(i–iii) show, respectively the accelerometer and gyroscope data from waist, thigh and ankle sensors for standing, sitting, 5 leg raises while sitting, 5 sit-to-stands, lying down and sitting on the floor with legs outstretched. In these plots the accelerometer and gyroscope signals have been low-pass filtered with a second-order Butterworth filter with a 3 Hz cut-off, run both forwards and backwards to minimise phase distortions. These data were from the right side of the Participant 2 Day 2 dataset (see P2D2diary.txt). Boundaries on the graphs are indicative of the approximate transitions between individual activities.

Segment 1 from Fig. 3a(i–iii) shows the ‘standing’ data and as expected they show that the z-axis of the accelerometer measures 1 *g*, while the x and y-axes measure 0 *g*, as the person was not moving while standing upright. In Fig. 3a,b, the 5 leg raises are clearly reflected in the ankle sensors (Segments 3i), while smaller signals are observed in the waist and thigh. In the 5 sit-to-stands (Segments 3ii), the activity is clearly reflected in the waist and thigh sensors, while smaller signals are observed in the ankle sensors.

The sensor angles with respect to the vertical axis are shown in Fig. 3c(i–iii). These inclination angles were estimated from the inverse cosine of the acceleration due to gravity as measured on the z-axis. The inclination angles were 0° for all the sensors when the participant was in the upright ‘standing still’ position, as the sensors were all aligned with vertical through the first step in the alignment process. In comparison, when the participant was in the ‘sitting’ and ‘sitting on the floor with legs outstretched’ positions, the angle for the waist was about 25°–40° as the participant was leaning forward/backward and the angle for the ‘thigh’ was approximately 90° as the thigh came to a horizontal position. These two postures can be distinguished by using the ankle sensor (sensor 1 in Fig. 1b). For ‘sitting’, the ankle was around 10° as the legs were inclined/reclined.When the participant was in the ‘sitting on the floor’ position it could be expected that ankle would be horizontal, however, the ankle angle was approximately 110°. This may be related to a shift in the clothing relative to the body, or possibly that the participant let their leg relax into a comfortable position, resulting in the toes facing outwards. More generally, it is possible that movement of the clothing relative to the body could affect the quality of the data captured, however, we would still anticipate a relatively good correlation between the sensor and the body arising from wearing the clothes. Nevertheless, this would be an interesting and worthwhile topic to investigate further.

**Fig. 4:**
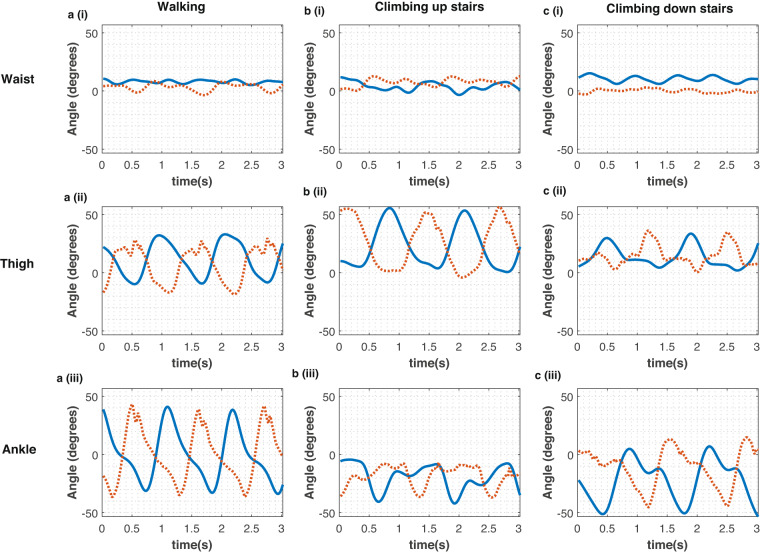
The “sensor to vertical” angles for the waist, thigh and ankle for dynamic activities for Participant 1 Day 1. Angles for walking, climbing up and down stairs, from the right leg (‘blue solid line’) and the left leg (‘red dotted line’) are shown.

To calculate the sensor-to-vertical angles for dynamic activities, rotation matrices were used. First, the data from each sensor was used to estimate quaternions using Madgwick’s algorithm^53^ (https://github.com/xioTechnologies/NGIMU-Software-Public, accessed on 21 September 2021) and the sensor-to vertical angles were estimated by calculating the angle between the forward pointing vector and the gravity vector (as described in^54^). The angles based on the lower-body sensors for walking, climbing up stairs and down stairs are shown in Fig. 4.

## Usage Notes

Corresponding MATLAB scripts are provided to access, reuse and visualize the data. The MAT files are readable not only in MATLAB but also in Python with packages such as ‘scipy’. Further, along with the data, video files and annotation files are given with a descriptive ‘readme’ file.

This dataset consists of 15 participant-days worth of data collected from 5 healthy adults, with each participant wearing clothes with sensors for between 1 and 4 days for 5–8 hours per day. One participant, P3, contributed just one day of data. The dataset lends itself well to posture and movement analysis and classification approaches such as the ones we have presented, however, the dataset may not generalise to a more diverse population and a larger catalogue of movements. Nevertheless, this dataset makes a worthwhile contribution in that it is, to our knowledge, the first published dataset from sensors embedded in loose clothing.

### .Supplementary information

Data Descriptor Worksheet 1

## Data Availability

Data and MATLAB scripts are available in figshare^46^. Further, CSV files of the MATLAB variables and Python scripts to read the MAT files directly are also available in figshare^46^
